# Different Sourced Extracellular Vesicles and Their Potential Applications in Clinical Treatments

**DOI:** 10.3390/cells11131989

**Published:** 2022-06-21

**Authors:** Leila Bahmani, Mujib Ullah

**Affiliations:** 1 Institute for Immunity and Transplantation, Stem Cell Biology and Regenerative Medicine, School of Medicine, Stanford University, Palo Alto, CA 94304, USA; leila.bahmani6508@yahoo.com; 2 Molecular Medicine Department of Medicine, Stanford University, Palo Alto, CA 94304, USA

**Keywords:** Extracellular vesicles, stem cells, clinical application, drug delivery, cellular communication

## Abstract

Extracellular vesicles (EVs) include a heterogeneous group of natural cell-derived nanostructures that are increasingly regarded as promising biotherapeutic agents and drug delivery vehicles in human medicine. Desirable intrinsic properties of EVs including the ability to bypass natural membranous barriers and to deliver their unique biomolecular cargo to specific cell populations position them as fiercely competitive alternatives for currently available cell therapies and artificial drug delivery platforms. EVs with distinct characteristics can be released from various cell types into the extracellular environment as a means of transmitting bioactive components and altering the status of the target cell. Despite the existence of a large number of preclinical studies confirming the therapeutic efficacy of different originated EVs for treating several pathological conditions, in this review, we first provide a brief overview of EV biophysical properties with an emphasis on their intrinsic therapeutic benefits over cell-based therapies and synthetic delivery systems. Next, we describe in detail different EVs derived from distinct cell sources, compare their advantages and disadvantages, and recapitulate their therapeutic effects on various human disorders to highlight the progress made in harnessing EVs for clinical applications. Finally, knowledge gaps and concrete hurdles that currently hinder the clinical translation of EV therapies are debated with a futuristic perspective.

## 1. Introduction

Extracellular vesicles (EVs) are membranous nanosized packages secreted from virtually all cell types into the biofluids as vital mediators of intercellular communication [1]. These nanoparticles have been implicated in a wide range of physiological processes due to their ability to transmit biological signals including DNAs, RNAs, and proteins from a source cell to a recipient [2]. EVs can be categorized into microvesicles, exosomes, and apoptotic bodies [3]. EVs carry bioactive macromolecules that are reflective of their producer cell and can cause gene expression alterations and post-translational modifications in the target cells [4,5]. Exosomes also contain complex bio-cargos derived from their parental cell and are involved in cellular communication [6,7].

Over the last decades, synthetic drug delivery systems have been developed in order to substitute conventional drugs that are prone to high clearance rate, low bioavailability, unspecific targeting, and consequent off-target side effects [8]. Artificial nanocarriers improve the bioavailability and treatment efficacy of therapeutic molecules by protecting them from enzymatic degradation, increasing their concentration at the target tissue, and inducing sequential release while minimizing undesirable side effects [9,10,11,12,13,14]. Nevertheless, only a few synthetic delivery systems have been successfully translated from bench to bedside, due in part to their high production cost, low transfection efficacy, poor biocompatibility, and safety concerns in terms of immunogenicity [15,16]. The intrinsic potential of EVs to survive in body fluids, protect their biologically active cargo from in vivo degradation, and deliver it efficiently to their specific target with nearly no immunogenicity make them stronger candidates for therapeutic delivery compared to synthetic competitors [17,18].

Many pioneering and innovative reports demonstrated that EVs exhibit a similar influence as their parent cell and therefore can be directly utilized as alternatives to intact cells in a cell-free manner for therapeutic purposes [19,20,21,22]. The use of EV therapeutics, as substitutes for cell therapy, confers certain advantages such as reducing the risk of immune rejection and tumorigenicity resulted from cell transplantation [23]. Emerging evidence also indicates that EVs isolated from different cell types such as mesenchymal stem cells, T cells, neural cells, and platelets, differ substantially from one another and exert distinct effects on their cellular targets [24]. This discrepancy stems from the fact that they contain diversified contents and present their unique molecular patterns. In recent years, an increasing number of preclinical studies has confirmed the therapeutic impact of different sourced EVs on diseases as various as degenerative diseases, immune and inflammatory disorders, and cancers [25,26,27,28]. These reports indicate promising progress in employing EVs as a new class of human therapeutics and natural delivery systems. Nevertheless, despite their medical potential demonstrated at the bench-top, the use of EVs as clinical platforms poses many technical issues due mainly to lack of standardization in EV isolation, detection, and handling protocols [29]. Standard and clinically compliant isolation, storage, and loading strategies are still required to achieve a scalable and reliable source of EVs without damaging their integrity and to ensure their successful translation to clinical interventions. 

With the rapid increase in employing different originated EVs, it is important to obtain a comprehensive understanding of their upsides and downsides and to discuss the novel applications of them in terms of their clinical utility. Therefore, in this review, after briefly describing EV biology and their intrinsic advantages over cell therapeutics and synthetic nanocarriers, we aim to summarize recent findings concerning specific features of different EVs, with reference to their therapeutic applications. We will also discuss major challenges that currently hamper the commercialization and clinical implementation of EV technology to highlight critical areas for future research.

## 2. Overview of EVs

### 2.1. Biogenesis

Extracellular vesicle is a general term commonly used to describe nanosized phospholipid bound packages of information that are ubiquitously released by myriad cell types into biological fluids including blood, urine, cerebrospinal fluid, ascites, saliva, and breast milk [30,31,32,33,34,35]. EVs comprise two main subclasses (microvesicles and exosomes) which are basically distinguished according to their size and biogenesis route [36,37].

Microvesicles which include a larger and more heterogeneous subgroup of EVs, range in size from 50 to 1000 nm and are directly generated from the cell membrane [38]. These vesicles are formed through outward budding in specific sites of the plasma membrane and then shed into the extracellular environment. Although the biogenesis and secretion processes of microvesicles have not been clearly defined yet, they appear to require an increased level of intracellular Ca^2+^ which provokes localized molecular rearrangements in lipid and protein composition of the membrane. Abscission of the cellular membrane is complemented by redistribution of payload biomolecules which are selectively recruited into microvesicles and culminates in the regulated secretion of microvesicles from the cell surface [39]. Microvesicle formation is a cytoskeleton-dependent process and different cytoskeletal elements including actin and myosin, their regulators and associated proteins such as RhoA GTPase and ROCK, are involved [40,41].

Unlike microvesicles, exosomes are generally characterized by their smaller dimension ranging from 30 to 100 nm and their intracellular biogenesis pathway. Exosomes are constitutively formed through an endocytic process, during which inward budding of endosomal membrane creates intraluminal vesicles (ILVs) which then further mature to generate multivesicular bodies(MVBs) [42]. Basically, two biogenesis pathways for exosomes have been identified, the endosomal sorting complexes required for transport (ESCRT)-dependent and ESCRT-independent mechanisms. ESCRT is an intricate protein complex composed of four separate subunits (ESCRT-0 to ESCRT-III) that work cooperatively for exosome generation [43]. In an ESCRT-dependent manner, first, ubiquitinated proteins on the endosomal membrane are recognized by ESCRT-0 which then interacts with ESCRT-I and ESCRT-II to initiate inward budding. Next, ESCRT-III is recruited to constitute the total complex that cleaves the buds to form MVBs [44]. In the final step, MVBs fuse with the cell membrane releasing exosomes into the extracellular space. Certain types of biomolecules such as ceramides and tetraspanins play pivotal roles in an ESCRT-independent manner of exosome generation. Ceramides which are formed through hydrolytic elimination of phosphocholine, are negatively charged and can induce negative curvature of the membrane in order to generate membrane subdomains and thereby promote domain-induced budding [45]. Tetraspanins such as CD9, CD81, and CD82 are also reported to be involved in cytoskeletal remodeling, microdomain formation, and eventual exosome cargo sorting [6,46]. Due to lack of specific markers for EV subpopulations and their overlapping features regarding size, biological functions, and composition, nomenclature of different EV subtypes is problematic. Therefore, in the subsequent sections of this review both subgroups are considered together by using the general term ‘EVs’.

### 2.2. Biological Applications

**Figure 1:**
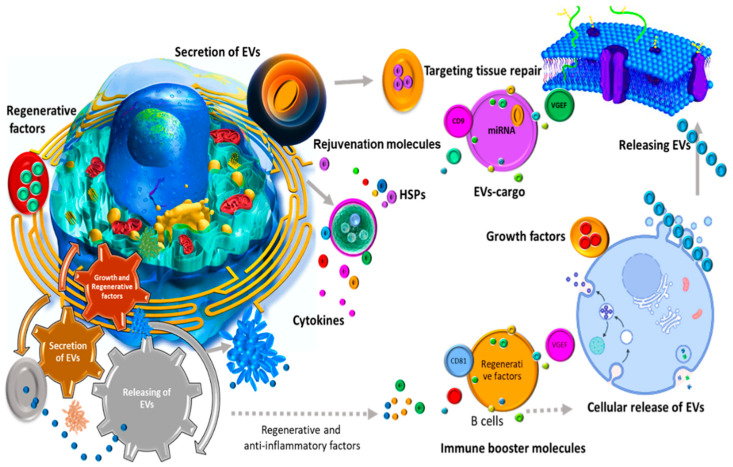
EVs play contrasting roles in normal physiology and pathological communication.

Despite initially being thought of as a removal mechanism of cell waste products, it has been subsequently suggested by a substantial body of evidence that EVs play indispensable roles in both normal physiology and pathological communication (Figure 1) [47,48,49,50,51]. Research on EVs shed by antigen presenting cells (APCs) such as B lymphocytes and dendritic cells showed that these nanovesicles are capable of inducing T cell-mediated immunity [52]. Developing this immune-activating potential of EVs opens up novel opportunities to treat various diseases as important as cancers [53]. Beyond their immunogenic capacity, EVs are involved in cell–cell communications and several landmark studies highlighted their potential to transfer encapsulated payloads including mRNAs, miRNAs, and proteins with the intent to activate downstream signaling pathways in the recipient cells [54,55]. Moreover, in the nervous system, EVs have been reported to enhance myelin formation, neurite growth, and nervous cell survival, and thus are able to contribute positively to tissue repair and neural regeneration [27,56,57]. Recently, it has been also observed that oviductal EVs fused with the membranes of sperm’s head and mid-piece can improve function and fertilization [58]. Interestingly, EVs from child gut microbiota seem to be necessary for their bone homeostasis and strength [59].

EVs participate in cell proliferation [60,61,62], and are known for their role in neurodegenerative disorders [63,64], and interaction with the immune system [65,66]. Considering the key role of certain EVs in biological processes, there is now a growing focus on developing such EVs as novel targets of therapeutic interventions [67].

### 2.3. Therapeutic Benefits

#### 2.3.1. EV Benefits Compared to Cell-Based Therapeutics

For years, cell-based therapies have been the main focus of various experimental studies and phase I to III clinical trials with the aim of replacing dysfunctional or dead cells with functional live ones and modifying the microenvironment of damaged tissue to prevent further damage and to activate reparative pathways [68,69,70]. The possible underpinning mechanism for therapeutic effects of transplanted cells is that these cells can reach their expected target site, engraft into damaged tissue, and then improve and/or recover its physiological function. Despite encouraging results obtained from preclinical and clinical studies, outstanding obstacles including the requirement of a large number of cells, limited homing and migratory capacity, high malignant transformation rate, possible entrapment in lungs and subsequent elimination from blood vessels, risk of vascular obstruction and immune rejection still prevent clinical translation of cell-based approaches at the desirable level [22]. On the other hand, increasing lines of evidence indicate that therapeutic effects of transplanted cells are mediated by their secretomes namely EVs and supports the hypothesis that EV therapy can be a rational alternative to intact cell transplantation [20,71]. EV-based therapeutics may confer several advantages in contrast to cell-based strategies (Table 1). EV transplantation does not evoke immune responses and thereby there is no need for immunosuppressive agents in acellular applications [48,72]. Based on accumulating studies reporting similar and even superior therapeutic properties of EVs in comparison with their parental cell, theses nanovesicles have promising potential to be used for the treatment of several human diseases such as heart diseases, neurodegenerative disorders, cancers, and even COVID-19 pneumonia [73,74,75,76].

#### 2.3.2. EV Benefits Compared to Synthetic Nanocarriers

Over the past few decades, various synthetic drug delivery vehicles have been developed and exploited in order to reduce renal elimination of drug molecules, improve site-specific targeting and advance simultaneous multidrug delivery [77,78]. Despite recent advancements in the field of synthetic viral and non-viral delivery systems, many hurdles limit their medical efficacy and prevent them from reaching clinical trials. For example, synthetic nanostructures are recognized by the human immune system and results in various immune responses from allergic reactions to final immune rejection [12,79]. Moreover, there are several challenges regarding improving their biocompatibility and transfection efficiency [80]. An additional drawback of using synthetic delivery systems is that the development and optimization of smart and multifunctional vehicles could be highly complex and costly [81]. In this respect, EVs have been recently considered as prospective natural delivery tools that can potentially tackle issues related to synthetic nanocarriers. These endogenous transporters possess inherent unique features such as non-immunogenic delivery, biocompatibility, and source availability that qualify them to substitute the current delivery tools in use [82,83,84,85]. Indeed, EVs are capable of avoiding the phagocytic system and surviving in circulation while carrying therapeutic materials. Specific lipid and protein composition of EV membrane also makes them capable of targeting and interacting with certain markers on the surface of target cells [82,83]. Overall, EVs with these favorable properties have attracted increasing interest as promising drug delivery systems in the world of human medicine (Table 1).

## 3. Different Sourced EVs and Their Therapeutic Potential

The observation that conditioned media from cultured cells preserves the majority of cell therapeutic efficacy along with the growing studies demonstrating the essential participation of EVs to the protective effects of cell administration, contribute to the rationale for developing EV-based strategies as a novel class of acellular treatment options [86,87,88]. Since EVs isolated from different cell sources appear differently in terms of clinical potential and exhibit a unique tendency to cure certain disorders based on their specific properties and signaling molecules, there is now a growing interest in exploring different sourced EVs for their therapeutic utility (Table 1).

### 3.1. Mesenchymal Stem Cell-Derived EVs

Mesenchymal stem cells (MSCs) include multipotent stem cells which are capable of self-renewal and differentiation to a huge variety of cell lineages such as osteocytes, myocytes, adipocytes, chondrocytes, and neurocytes [89,90,91,92]. MSCs can be found in different parts of the body and their principal function is considered to be dead cell replacement through migration to damaged tissue and differentiation into the desired cell type which ultimately results in physiological homeostasis [93,94]. Therapeutic efficacy of MSCs in many pathological conditions such as autoimmune, neurodegenerative, liver, cardiac and kidney diseases was first attributed to their differentiation potential. However, it then became clear that only around 1% of transplanted MSCs reach the target area and therefore their secretome was proposed to account for the beneficial properties of MSCs [95,96]. Since none of the MSCs identified released factors was able to efficiently mediate their treatment applications, it was consequently suggested by many researchers that MSC-derived EVs are the main therapeutic mediators [97,98]. MSC-EV bioactive cargo contains not only proteins and RNAs associated with EV biogenesis, trafficking, and fusion, but also those related to self-renewal and differentiation pathways in MSCs [99,100]. Although, all MSCs are prolific producers of EVs, bone marrow-, umbilical cord- and adipose tissue-derived MSCs are the most common sources for MSC-EVs required in preclinical and clinical studies [101,102,103].

Over recent years, bone marrow stem cell-derived EVs (BMSC-EVs) used therapeutically in preclinical and clinical trials have shown considerable protective properties across several pathological conditions. For instance, BMSC-EVs ameliorated osteoarthritis through inducing chondrocytes to produce type II collagen and inhibiting TNF-α mediated inflammatory pathway [104]. Another study showed that tendon damages could be recovered using BMSC-EV administration which caused a decrease of inflammatory and apoptotic cells and an increase in the tendon progenitor population [105]. It was also discovered that intravenous injection of BMSC-EVs has therapeutic effects on acute kidney injuries caused by prolonged exposure to heavy metals [106]. In a mouse model of diabetic nephropathy, EVs could inhibit fibrosis progress via fibrogenic gene downregulation [107]. In a study of graft versus host disease (GVHD) mice, systemic infusion of nanovesicles isolated from bone marrow stem cells could prolong mice survival and reduce GVHD-related pathology [108]. This therapeutic efficacy resulted from suppressing the differentiation of naïve T cells into effector T lineages while maintaining the population of regulatory T (Treg) cells. In the aspect of myocardial infarction, BMSC-EVs improve myocardial recovery through a GATA-4-dependent mechanism [109]. BMSC-EVs have also the ability to slow cell senescence and prolong life span in culture and in vivo [110]. It is worth noting that the MSC-EV (particularly BMSC-EVs) potential to alleviate inflammatory response has recently raised the possibilities for their development as promising COVID-19 treatment agents [111,112,113].

The therapeutic efficacy of umbilical cord stem cell-derived EVs (UCSC-EVs) has been also investigated extensively in many incurable illnesses and at preclinical and clinical levels. In nerve injuries, for example, EVs isolated from the umbilical cord promoted nerve regeneration and motor function in the damaged areas [114]. UCSC-EVs are also suggested as possible therapeutic tools for bone loss since they can significantly improve microRNA-3960-mediated osteogenic differentiation of BMSCs [115]. Regarding liver injuries, studies revealed the positive contribution of UCSC-EVs to the reduction of hepatic inflammation, collagen deposition, oxidative stress, and apoptosis in liver fibrosis [116]. At the clinical level, UCSC-EV treatment showed positive effects and improved kidney function in grade III-IV kidney disease patients [117]. Although UCSC-EVs exhibit nearly similar therapeutic potential across a range of disorders in comparison with BMSC-EVs, they hold unique medical properties for infant and gynecological disorders. It has been proved by two separate scientific endeavors that UCSC-EVs play a neuroprotective role in perinatal brain injuries [118,119]. Additionally, exosomes isolated from human umbilical cord stem cells showed considerable treatment effects on ovarian granulosa cell apoptosis via Bcl-2 and caspase-3 upregulation [120]. A higher proliferation and EV secretion rate of umbilical cord stem cells may also make them a better choice than bone marrow and adipose stem cells for future therapeutic purposes [121].

Adipose tissue is another common source of MSCs and adipose tissue stem cell-derived EVs (ADSC-EVs) have been shown to be as effective as those of the other two sources in terms of therapeutic utility across most explored diseases. Therefore, ADSC-EVs can be considered as a preferred choice when stem cell and thereby EV isolation from other sources is difficult or in case they show distinct therapeutic effects [122]. In a preclinical study, it was shown that the anti-inflammatory property of ADSC-EVs attenuates allergic asthma via reducing the interleukin(IL)-5 level in lung tissue [123]. The neuroprotective potential of ADSC-EVs has also been proved by several elegant studies. In one of them, ADSC-EV administration could modulate the apoptotic function of mutant Huntingtin aggregates by which Huntington’s disease (HD) phenotype was improved [124]. In an Alzheimer’s disease (AD) mouse model, it was also revealed that ADSC-EVs reduced beta-amyloid mediated neuronal death and prevented disease progression [125]. Moreover, intravenous injection of murine ADSC-EVs lessened spinal cord inflammation and demyelination in an experimental autoimmune encephalomyelitis (EAE) mouse model [126]. In the aspect of wound healing, experimental findings pointed out that systemic administration of ADSC-EVs, particularly in the early stages, could reduce scar formation by increasing the gene expression of collagen type III, collagen type I, PCNA, cyclin-1, and N-cadherin or by modifying the ratio of collagen III and I [127,128]. Recently, a study that tried to use ADSC-EVs to modulate osteoarthritis, highlighted the potential of these EVs to affect the gene expression and cytokine release patterns of chondrocytes and synoviocytes by which they can reduce IL-1ß-mediated inflammation [129].

### 3.2. Immune Cell-Derived EVs

Based on their parental cell type and state, immune cell-derived EVs are capable of inducing either immune-stimulatory or immune-inhibitory responses and therefore can play dual roles in physiological and pathological processes. Ample evidence has been found that EVs isolated from different immune cells including macrophages, dendritic cells (DCs), T cells and natural killer (NK) cells exhibit distinct functions. For instance, CD8^+^ T cell-derived EVs exert immuno-activating properties and are being used in several research works with the intent to shrink tumor growth, whereas Treg cell-derived EV administration is emerging as a promising strategy for transplantation tolerance because of the immunomodulatory effects [130,131]. In terms of EVs isolated from different cell states, it has been also unraveled that injection of EVs from mature DCs activates effector T cell responses, while immature DC-derived EVs induce tolerogenic pathways [132]. The ability of these immune active EVs to regulate immune responses through presenting antigens, activating NK and T cells responses, or inducing Treg cell differentiation, makes them ideal candidates for prospective therapeutic interventions [133].

Over recent years, DC-derived EVs have been extensively explored as antigen delivery tools for anti-cancer treatments. Phase I and II trials that used EVs isolated from DCs of metastatic melanoma and non-small cell lung cancer patients for cancer therapy have proven that using immune cell-derived EVs in clinical applications is feasible and safe, although they failed to activate sufficient immune responses [134,135]. Various modification strategies were then exploited to enhance the immune-stimulatory properties of DC-derived EVs leading to stronger immune responses. For example, co-delivery of tumor peptides with α-galactosylceramide can induce an adaptive anti-tumor immunity in a mouse model of melanoma [136]. It was also demonstrated that EVs secreted by bone marrow DCs elicit improved anti-tumor activity upon Toll-like receptor 3 (TLR3) stimulation in DCs [137]. Additionally, in a mouse model of hepatocellular carcinoma, DC-EVs expressing tumor antigen α-fetoprotein and transfected by lentivirus could suppress tumor progression by increasing the percentage of CD8^+^ T cells and reducing the number of Treg cells in the tumor microenvironment [138]. The therapeutic efficacy of DC-derived EVs is not limited to cancer therapy and it is suggested that low dose release of such EVs can potentially induce immune responses against HIV-1 infection [139]. Besides, systemic delivery of DC-derived EVs could mediate CD4^+^ T cell activation and improve cardiac function post-myocardial infarction in mice [140].

Since NK-derived EVs, like their parental cell, contain lytic proteins such as perforin, FasL, granzyme A and B, it has been suggested that they can also serve as potential cancer therapeutics [141]. Anti-tumor characteristics of NK-EVs were confirmed by several in vitro and in vivo experiments that demonstrated the toxicity of such EVs to human glioblastoma, melanoma, and breast carcinoma [141,142,143]. As mentioned before, CD8^+^ T cell-derived EVs are another population of immune active EVs that can be exploited in cancer treatments by reducing cancer MSCs and thereby inhibiting tumor progression [131].

On the other hand, the immunosuppressive features of immune cell-derived EVs have raised a great possibility for them to be used as therapeutic agents in transplantation tolerance and autoimmune disease treatment.

Reduced expression of MHC molecules and co-stimulatory factors on immature DCs can create their immune-suppressive ability and their isolated EVs seem to possess similar immune-modulatory features [132]. Two separate studies reported that immature allogenic DC-EVs could prevent graft rejection and prolong survival in animal models of cardiac and intestinal transplantations [144,145]. However, their immune-suppressing effects were enhanced by combination with transplantation drugs. Treg cells are in charge of suppressing excessive immune responses by inhibiting effector T cell differentiation and proinflammatory cytokine production [146]. Their secreted EVs, therefore, hold great potential to act as immunomodulators in therapeutic applications. It was reported that Treg-derived EV administration in a rat model of kidney transplantation could postpone graft rejection and extend its survival using certain microRNAs and inducible nitric oxide synthase [147].

In the context of autoimmune disorders, DC-derived EVs containing small interfering RNA (siRNA) against glyceraldehyde-3-phosphate dehydrogenase could effectively inhibit the expression of this gene in all neural cells including neurons, microglia, and oligodendrocytes in a mouse model of AD [148]. In addition, various engineering strategies can be applied to improve the immune-inhibiting capacity of immune cell-derived EVs. For example, EVs isolated from DCs genetically modified to express IL-4 or indoleamine-pyrrole 2,3-dioxygenase can ameliorate autoimmune inflammatory diseases in murine models [99,149].

Overall, immune cell-derived EVs have been drawing increasing attention due to their favorable properties including immune-suppressing and immune-stimulatory potential. They can be also isolated from the individual patient for autologous treatments and various studies reported their superior efficacy over immune cell-based therapies as well [150]. However, as notified before, natural immune cell-derived EVs are incapable of inducing therapeutic effects at the desired level and need to be engineered or used in combination with other drugs to improve their efficacy. Furthermore, since immune cell-derived EVs exert contrast effects over their maturation and in different physiological states, it would be crucial to optimize immune active EV-based therapies according to different medical purposes.

### 3.3. Blood Cell-Derived EVs

Over the last decade, there was a massive upsurge in studies investigating the therapeutic applications of blood cell-derived EVs (BC-EVs) due largely to their exceptional advantages over EVs originated from other cell types. BC-EVs are conveniently accessible from human blood and since blood cells are the most abundant cells in the human body, scalable amounts of BC-EVs can be provided at low cost [151,152,153,154]. Red blood cells and platelets are the most widely used sources of BC-EVs in preclinical and clinical studies [155,156].

**Figure 2:**
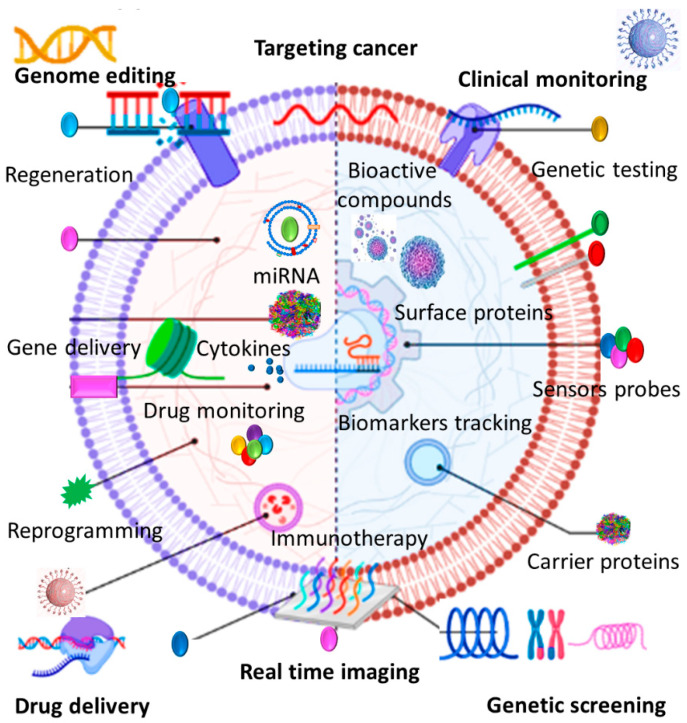
EVs can be involved in cancer therapy as diagnostic markers, therapeutics, and drug delivery tools.

Red blood cells (RBCs) represent a great source of EVs due in part to their availability, abundance, extended life span, and lack of intracellular DNA [157]. RBCs can be easily obtained from patients’ blood or blood banks and there are 5 billion RBCs/mL of human blood. This can dispel the need for cell expansion in vitro and obviate the risk of mutation during cell passaging [155]. Surface proteins on the RBC membrane such as CD47, CD59, and CR1 prevent immune clearance pathways and prolong their circulation time in the bloodstream [158,159]. Recent studies demonstrate that RBC-EVs can be considered as robust markers and delivery tools particularly in cancer diagnosis and treatment since they hold the same advantages as their producer cells (Figure 2). RBC-EVs are continuously released over the 120-day life span of RBCs and because of their inherited membrane proteins; they exhibit a longer elimination half-life [160]. More importantly, EVs isolated from mature RBCs do not contain nuclear and mitochondrial DNA and therefore there is no risk of horizontal gene transfer in RBC-EV-based therapeutics [161]. Therapeutic safety and efficacy of RBC-EVs have been proved by different studies that employed them as RNA delivery platforms in solid and liquid tumor treatments. Intra-tumoral injection of antisense RNA-loaded RBC-EVs could efficiently inhibit breast tumor growth and systemic administration of RBC-EVs and transferring microRNA-125b antisense oligonucleotide significantly suppressed the microRNA expression level and dampened acute myeloid leukemia cell growth [162]. In comparison with synthetic nanocarriers, it has been proved that RBC-EVS showed a superior targeting capacity for lipophilic drug delivery to lung carcinoma cells [163]. RBC-EVs can be also applicable for iron oxide delivery into bone marrow MSCs for cellular magnetic resonance imaging [164]. Given their abundance, availability, extended half-life, and safer profile, RBC-EVs present several advantages over other sourced EVs. Nonetheless, there remain critical challenges in terms of their clinical translation. Proteomic assays have demonstrated that the protein composition of RBC-EVs generated under different stimulating and storage conditions may differ considerably [165]. Moreover, increased levels of RBC-EVs can contribute to coagulation, thrombosis, and dysregulate nitric oxide and oxygen homeostasis [166]. Therefore, more in-depth therapeutic assessments are required to be conducted in future scientific endeavors to explore the true impact of different RBC-EVs and optimize their efficient dose.

Platelets are another primary source of EVs in the bloodstream and studies estimate a concentration of 11,500 platelet-derived EVs (P-EVs)/µL in healthy plasma [167]. P-EVs are generated by platelets upon stimulation and their secretion remarkably increases during chronic inflammations and different disease states [168,169,170]. Due to their considerable roles in biological and pathological conditions including coagulation, inflammation, angiogenesis, and tumor progression, P-EVs have gained increasing attention to be used in next-generation therapeutic strategies [21,171,172,173]. In vascular injuries, for example, P- EVs enhanced angiogenesis and endothelial restoration [174]. P-EVs filled with growth factors of Akt and Erk pathways could also induce angiogenesis and promote neural stem cells proliferation and differentiation after cerebral ischemia [175]. Further, proliferation, survival and osteogenic differentiation of bone marrow MSCs were boosted when treated with P-EVs [176]. Platelet-rich plasma (PRP) is a concentration of platelets which is widely used to accelerate the healing of injured tissues. Since P-EVs show similar angiogenic and proliferative effects as PRP, there is now a growing interest in P-EV-based therapeutics to trigger tissue repair and subsequently treat chronic wounds [156]. It has been demonstrated in several studies that PRP-derived EVs can induce wound healing through PI3K-Akt and MAPK-Erk pathways and via yes-associated protein (YAP) activation, the same as PRP [156]. Two separate in vivo studies reported that EVs derived from human PRP prevented apoptosis and promoted re-epithelization in rat models of osteonecrosis and diabetes, respectively [156,177]. Frozen P-EVs are likely to provide a better trauma and bleeding treatment option than PRP in conflicts and wars where PRP half-life and storage can be truly problematic [178].

Due to the prominent role of P-EVs in cancer pathology, novel treatments that are designed to curb P-EV secretion stimulated by cancer cells, might replace strategies that disrupt the entire function of platelets [173].

Taken together, all these observations suggest that P-EVs can be considered as potential alternatives to current expensive stem cell therapies and cancer treatments. Nonetheless, there remain considerable ambiguities and uncertainties that should be given more careful attention. As notified, platelets need to be activated prior to EV secretion and depend on their stimulant; released EVs are highly heterogeneous in terms of biological content and function. Furthermore, the roles of P-EVs in tissue repair and cancer pathology are inextricably intertwined. Considering these points, it is prudent to further investigate the underlying function of different P-EVs and develop more careful isolation techniques before their clinical usage.

### 3.4. Neural Cell-Derived EVs

Neural cells including neurons, astrocytes, oligodendrocytes, and microglia release EVs as a vital means of neural communications [179,180]. While neural cell-derived EVs (N-EVs) constitute an essential part of neural circuits and are involved in neural cell proliferation, differentiation, and synaptogenesis, growing evidence demonstrates that these nanostructures are also implemented in the propagation of various central nervous system (CNS) diseases [181,182]. 

N-EVs are widely distributed in biofluids such as blood and urine and their number and content reflect the status of their producer cell and tissue [183]. There has been recently a wave of enthusiasm for developing circulating N-EVs as neurodegenerative disease diagnostic and prognostic biomarkers which can be simply measured by inexpensive and noninvasive blood tests [184,185]. More importantly, introducing non-pathogenic N-EVs carrying therapeutic molecules or drug compounds into the brain of patients may hold promising potential for treating incurable CNS diseases in the near future [186,187,188]. In vivo experiments have clearly demonstrated that intracerebral administration of neuron-derived EVs reduced toxic fibrils and ameliorated Aß pathology in AD transgenic mice [189]. Research has also illustrated that incorporation of a specific neuronal exosomal microRNA into astrocytes can alter their gene expression pattern to promote neural plasticity and functional recovery after stroke [190,191]. Neuronal EVs are also able to induce neural differentiation of ADSCs in a SNAP25, miR-9, and miR-132-dependent manner, suggesting that co-treatment of ADSCs and N-EVs may reduce peripheral nerve degeneration after injury [192]. In a recent study, the transfer of a therapeutic microRNA targeting mutant Huntingtin gene (*HTT*) within neuronal EVs caused efficient *HTT* decrease in vitro and in vivo in the brain of HD animal models [193].

Together, due to their dual roles under normal and abnormal conditions, N-EVs have the potential to be used as CNS disease biomarkers, drug targets, and therapeutic options. However, several technical limitations ahead for their clinical translation remain to be overcome. Cerebrospinal fluid (CSF) collection for selective isolation of N-EVs is quite challenging because of the invasive nature of the procedure and the small size of the resultant samples [194,195]. To tackle this issue, peripheral blood has been suggested to purify N-EVs. Unfortunately, this approach imposes additional challenges to ensure clinical accuracy due to the lack of N-EV specific surface markers and the possible introduction of contaminating factors [196]. Indeed, N-EVs may undergo physiologic drift during migration from CNS fluid to peripheral blood, and blood collection can cause the artificial creation of platelet- and immune cell-derived EVs as well. In consequence, important questions are raised as to whether blood purified EVs are truly neural-derived and can be considered reliable for clinical application in neurology.

## 4. Current Challenges and Future Directions

Despite numerous promising results in preclinical and mere clinical studies, many challenges remain and, therefore, more in-depth investigations are needed to accelerate the clinical translation of EV-based therapeutics. Due to the dual roles of EVs in natural processes and the pathology of several diseases, there remain outstanding questions regarding the accurate characterization of pathogenic and non-pathogenic EVs [197,198,199,200]. A greater understanding of EV biogenesis, surface markers, cargo, uptake, and functions will allow us to precisely distinguish different EVs and to develop therapeutics completely void of undesirable effects. Furthermore, more detailed information about specific markers and functions of EVs from different parental cells can be enormously helpful to establish criteria for utilizing the optimal EV population with the highest efficacy in therapeutic and diagnostic applications.

The future of EV therapies is dependent on high-purity isolation and large-scale production of EVs. Today, many sample collection and EV isolation methods are ignored due to damage, interference, non-specific isolation, or inadequate production [201]. Going forward, it is necessary to develop standard methodologies of sample procurement, collection, processing, and storage with step-by-step instructions specialized for each EV source to minimize the variability of results over different laboratories and speed up the sampling procedures. There is also a significant need for designing novel isolation technologies and analytical techniques to isolate high-purity homogenous EV populations and thereby ensuring specific and more efficient EV-based therapies.

Addressing the issues regarding the engineered EVs could be another goal of future scientific efforts. Currently, the drawbacks of direct and indirect engineered EVs including poor EV recovery rate, limited cargo loading capacity, inadequate isolation yield, along with safety concerns, hinder moving of EV-based products towards clinical trials. In the current decade, these limitations need to be removed to develop the next generation of EV therapies.

## 5. Conclusions

EVs comprising microvesicles and exosomes can be released by nearly all cell types into the biofluids. EVs are central elements in cell-to-cell communications by which they contribute to various physiological contexts and pathological states including angiogenesis, inflammation, tissue regeneration, neural communication, tumor progression, and cancer cell migration. Due to their advantageous features such as effective packaging, biocompatibility, targeted delivery, and non-toxicological profile, EV-based products are being increasingly considered as practical alternatives to cell-based therapies and synthetic nanocarriers. The broad and increasing interest in the isolation of different sourced EVs has also opened up the possibility to optimize EV-based therapeutics for each specific clinical purpose. EVs released by different cell lineages hold distinct characteristics and thereby can be applied in either diagnostic or therapeutic applications. Despite the enormous therapeutical potential, EV technology is still in its infancy and there remain significant needs for standardized techniques of EV isolation, characterization, storage, and manipulation. Therefore, in order to fully translate EV-based therapies to clinical settings, in the future these hurdles must be overcome.

## Highlights

- Extracellular vesicles (EVs) are promising natural nanoscale delivery platforms.
- Desired cargos can be loaded into EVs via multiple methods to enhance therapeutic applications.
- EVs protect their cargo from degradation, and enzymatic digestion.
- EVs do not provoke the immune system and are capable for specific targeting in nanomedicine.

## Figures and Tables

**Table 1:** Advantages and disadvantages of EV-based therapeutics, stem cell therapies and synthetic nanocarriers.

	Advantages	Disadvantages
Extracellular vesicles Stem cells	- Cell-free agents - No risk for malignant transformation - Nano-scale size - Minimal risk of lung entrapment and vascular obstruction - Released by all cell types - Detected in all biological fluids - Availability as various diseases biomarkers - Ability to avoid phagocytosis and enzymatic degradation - Stability in body fluids - Ability to cross biological barriers including blood–brain barrier - Endogenous entity - Low immunogenicity - Safe in clinical trials - Ability for specific targeting - Potential for bioengineering - Suitable for (multi) drug delivery - Beneficial toxicity profile - herapeutic efficacy for several human diseases	- Lack of unified nomenclature system - Lack of standardized isolation, characterization and manipulation methods - Disability to differentiate - Difficulty of large-scale production - Limited isolation strategies for high yield - Insufficient knowledge of mechanism of action - Risk of viral infection, tumor progression, and neurodegenerative diseases - Short half-life in circulation - Insufficient clinical evaluation studies - Low drug loading efficiency
	- Intact alive cells - Potential to differentiate into various cell lineages - Ability of tissue regeneration - Well-described isolation, expansion and manipulation techniques - Potential for bioengineering - Approved clinical efficacy for treatment of certain disorders	- Risk of malignant transformation - Risk of lung entrapment and vascular obstruction - Limited homing and targeting potential - Risk of immune rejection - Insufficient therapeutic efficacy for some human - disorders - Risk of decreased viability and altered properties during cryopreservation
Synthetic nanocarriers	- Ease of large-scale production - high loading efficiency - Ability to deliver a wide variety of drugs - Protection of drugs from enzymatic degradation - Different route of administration - Well-defined uptake and drug release mechanisms - Ability for sequential drug release - Potential of development for multi-drug delivery	- Risk of toxicity - Low targeted delivery efficiency - High risk of clearance - Risk of immunogenicity - Expensive modification for multi-drug delivery - Complicate bioengineering and difficult handling

## Data Availability

Not applicable.

## Abbreviations

Extracellular vesicles (EVs), Endosomal sorting complexes required for transport (ESCRT), Human immunodeficiency virus (HIV), Mesenchymal stem cells (MSCs), Bone marrow stem cell-derived EVs (BMSC-EVs), Graft versus host disease (GVHD), Regulatory T cell (Treg cell), umbilical cord stem cell-derived EVs (UCSC-EVs), adipose tissue stem cell-derived EVs (ADSC-EVs), Interleukin(IL), Huntington’s disease (HD), Alzheimer’s disease (AD), Experimental autoimmune encephalomyelitis (EAE), dendritic cells (DCs), natural killer (NK), Toll-like receptor 3 (TLR3), small interfering RNA (siRNA), blood cells-derived EVs (BC-EVs), Red blood cells (RBCs), platelet-derived EVs (P-EVs), neural cell-derived EVs (N-EVs), central nervous system (CNS), Parkinson’s disease (PD), Amyotrophic lateral sclerosis (ALS), Huntingtin gene (HTT), Cerebrospinal fluid (CSF).
